# A Multi-Center, Open-Label, Randomized Controlled Trial to Evaluate the Efficacy of Convalescent Plasma Therapy for Coronavirus Disease 2019: A Trial Protocol (COVIPLA-RCT)

**DOI:** 10.3390/life12060856

**Published:** 2022-06-08

**Authors:** Noriko Tomita, Sho Saito, Junko Terada-Hirashima, Ayako Mikami, Yukari Uemura, Satoshi Kutsuna, Hidetoshi Nomoto, Kyoko Fujisawa, Maki Nagashima, Mari Terada, Shinobu Ashida, Shinichiro Morioka, Masahiro Satake, Akira Hangaishi, Tomiteru Togano, Katsuyuki Shiratori, Yuki Takamatsu, Kenji Maeda, Norio Ohmagari, Wataru Sugiura, Hiroaki Mitsuya

**Affiliations:** 1 Center for Clinical Sciences, Center Hospital of the National Center for Global Health and Medicine, Tokyo 162-8655, Japan; ntomita@hosp.ncgm.go.jp (N.T.); amikami@hosp.ncgm.go.jp (A.M.); yuemura@hosp.ncgm.go.jp (Y.U.); materada@hosp.ncgm.go.jp (M.T.); wsugiura@hosp.ncgm.go.jp (W.S.); 2 Disease Control and Prevention Center, Center Hospital of the National Center for Global Health and Medicine, Tokyo 162-8655, Japan; ssaito@hosp.ncgm.go.jp (S.S.); kutsuna@hp-infect.med.osaka-u.ac.jp (S.K.); hnomoto@hosp.ncgm.go.jp (H.N.); kfujisawa@hosp.ncgm.go.jp (K.F.); mnagamatsu@hosp.ncgm.go.jp (M.N.); sashida@hosp.ncgm.go.jp (S.A.); shmorioka@hosp.ncgm.go.jp (S.M.); nohmagari@hosp.ncgm.go.jp (N.O.); 3 Department of Infection Control and Prevention, Osaka University Hospital, Osaka 565-0871, Japan; 4 Central Blood Institute, Japanese Red Cross Society, Tokyo 135-8521, Japan; m-satake@jrc.or.jp; 5 Department of Hematology, Center Hospital of the National Center for Global Health and Medicine, Tokyo 162-8655, Japan; ahangaishi@hosp.ncgm.go.jp (A.H.); ttogano@hosp.ncgm.go.jp (T.T.); 6 Labotatory Testing Department, Center Hospital of the National Center for Global Health and Medicine, Tokyo 162-8655, Japan; kshiratori@hosp.ncgm.go.jp; 7 Department of Refractory Viral Infections, National Center for Global Health and Medicine Research Institute, Tokyo 162-8655, Japan; ytakamatsu@ri.ncgm.go.jp (Y.T.); kmaeda@ri.ncgm.go.jp (K.M.); hmitsuya@hosp.ncgm.go.jp (H.M.)

**Keywords:** plasma therapy, coronavirus disease 2019, COVID-19, SARS-CoV-2, viral load

## Abstract

Background: Coronavirus disease 2019 is a global public health concern. As of December 2020, the therapeutic agents approved for coronavirus disease 2019 in Japan were limited to two drugs: remdesivir, an antiviral drug, granted a Special Approval for Emergency on 7 May 2020, and dexamethasone, which has an anti-inflammatory effect. The aim of this study is to evaluate the efficacy of convalescent plasma collected from donors who recovered from coronavirus disease 2019. Methods: This is an open-label, randomized controlled trial comprising two groups: a convalescent plasma and a standard-of-care group. Plasma administered to patients with coronavirus disease 2019 randomized in the convalescent plasma group of this trial will be plasma that has been collected and stored in an associated study. Patients with a diagnosis of mild coronavirus disease 2019 will be included in this trial. The efficacy of convalescent plasma transfusion will be evaluated by comparing the convalescent plasma group to the standard-of-care group (without convalescent plasma transfusion) with respect to changes in the viral load and other measures. The primary endpoint will be time-weighted average changes in the SARS-CoV-2 virus load in nasopharyngeal swabs from day 0 to days 3 and 5. It is hypothesized that the intervention should result in a decrease in the viral load in the convalescent plasma group until day 5. This endpoint has been used as a change in viral load has and been used as an index of therapeutic effect in several previous studies. Discussion: The proposed trial has the potential to prevent patients with mild COVID-19 from developing a more severe illness. Several RCTs of convalescent plasma therapy have already been conducted in countries outside of Japan, but no conclusion has been reached with respect to the efficacy of convalescent plasma therapy, which is likely in part because of the heterogeneity of the types of target patients, interventions, and endpoints among trials. Actually, previous clinical trials on plasma therapy have shown inconsistent efficacy and are sometimes ineffective in COVID-19 patients with severe disease, which is due to unmeasured neutralizing antibody titer in the COVID-19 convalescent plasma. To improve this issue, in this study, we measure neutralizing activity of convalescent plasma before administration and provide the plasma with high neutralizing activity to the subjects. It is hoped that this study will further evidence to support the role of convalescent plasma therapy in COVID-19.

## 1. Background

Coronavirus disease 2019 (COVID-19) has spread and become a substantial public health concern worldwide. In addition, in Japan, as of 30 May 2022, the number of infected patients has reached 8.78 million or more through the first to sixth waves, and there is a concern over a continuation of the epidemic.

As of December 2020, the therapeutic agents approved for COVID-19 in Japan were limited to two drugs: remdesivir, an antiviral drug, granted a Special Approval for Emergency on 7 May 2020, and dexamethasone, which has an anti-inflammatory effect. In a global, randomized controlled clinical study (ACTT-1) sponsored by the National Institute of Health (NIH), the time to clinical improvement was shorter in the remdesivir group (10 d) by 5 d than in the placebo group (15 d) [1]. However, in an open-label controlled clinical study (SOLIDARITY trial) sponsored by the World Health Organization (WHO), the ratio of the risk of death in the remdesivir group relative to that in the standard of care group was 0.95 [2]. Consequently, the WHO does not recommend the use of remdesivir in its treatment guidelines [3]. In principle, it is indicated for patients with an oxygen saturation of ≤94% or those requiring supplemental oxygen, the introduction of extracorporeal membrane oxygenation (ECMO) or invasive mechanical ventilation. In a large, multi-center, randomized, open-label study in the United Kingdom (UK), mortality was lower in patients given dexamethasone than in those given the standard of care. The maximal therapeutic effect was observed in patients who required invasive mechanical ventilation at the time of randomization, in whom mortality was 29.0% in the dexamethasone group and 40.7% in the control group [4]. 

Convalescent plasma therapy was used in patients with Spanish flu, and a report suggests that its efficacy was demonstrated by an analysis of the patients given the therapy at that time [5]. More than 40 years ago, the therapy was reported to have decreased mortality in a randomized controlled study in patients with Argentine hemorrhagic fever [6]. Recently, convalescent plasma therapy has been used in patients with severe infection, such as avian influenza H5N1 [7] and Ebola disease [8], and those with coronavirus diseases similar to COVID-19, such as severe acute respiratory syndrome (SARS) [9] and Middle East respiratory syndrome (MERS) [10,11].

Several clinical studies of convalescent plasma therapy in patients with COVID-19 have been reported in China and the United States. In the former, a case series from China showed that clinical symptoms rapidly improved, and cures were achieved in all five patients who underwent convalescent plasma transfusion [12]. To date, three randomized controlled trials (RCTs) have been reported. In one RCT in China [13], convalescent plasma therapy was effective in patients with moderate disease who required supplemental oxygen but did not require mechanical ventilation. However, its efficacy was not shown in all patients or those with severe disease requiring mechanical ventilation. This trial was terminated before the planned number of patients was reached because it was conducted in the latter part of the epidemic in China, and the registration of patients decreased sharply in mid-trial. Therefore, it is possible that its efficacy in severe disease could not be shown because of the small sample size. Another RCT conducted on 81 hospitalized patients in Spain [14] showed that survival improved in patients who underwent convalescent plasma transfusion. The transfusion of the convalescent plasma was not effective in RCTs of patients with at least moderate disease in India [15] or Argentina [16]. However, the prevention of severe disease was demonstrated in an RCT where convalescent plasma was administered within 3 d after disease onset in patients with a high risk for severe disease, such as elderly patients and those with underlying diseases [17]. 

Based on these results, it is likely that convalescent plasma therapy is ineffective in patients with severe disease but most effective when the plasma with a high antibody titer is administered as soon as possible after disease onset. The inconsistent results of clinical studies on plasma therapy could be attributable to the neutralizing antibody titer (i.e., the extent of the potency to inhibit novel coronavirus infection) in the COVID-19 convalescent plasma administered. This value is unmeasured in most studies. Importantly, the intensities of neutralizing antibodies in the COVID-19 convalescent plasma greatly vary among individuals. That is, the neutralizing activity may be high in the plasma of one patient, whereas no neutralizing activity may be observed in the plasma of another. In addition, it has been reported that neutralizing antibodies are often decreased or disappear approximately 1 month after a high level of neutralizing antibodies is observed; a similar finding was confirmed in a study conducted by the Research Institute National Center for Global Health and Medicine [18].

In this trial, subjects will receive plasma with high neutralizing activity after it is determined whether the convalescent plasma from each donor can inhibit infection when susceptible living cells are exposed to infectious novel coronaviruses at the Research Institute National Center for Global Health and Medicine (directed by Hiroaki Mitsuya). Based on the above-mentioned facts, the objective of this trial is to evaluate the efficacy of convalescent plasma with high neutralizing activity collected from donors who have recovered from COVID-19. 

As potential risks and adverse events of the convalescent plasma, if a donor is affected by an unknown infection that is currently difficult to screen, a subject who has received plasma may acquire the infection. Although the donors will be interviewed according to the screening procedures used by the Japanese Red Cross Society, the risk of infection in this trial could be higher than that of standard blood transfusion because some screening criteria have been changed in this trial, allowing the acceptance of convalescent plasma from patients with COVID-19 or diabetes mellitus. In addition, adverse reactions, such as hypersensitivity, shock, anaphylaxis, and multi-organ failure, may occur because of immunization and antigen–antibody reactions. If a donor has been pregnant before or has undergone a blood transfusion, human leukocyte antigen (HLA) antibodies could be present in the blood product, which could increase the risk of transfusion-related acute lung injury (TRALI) after administration of the plasma product. However, persons who tested positive for HLA antibodies are excluded from the donors in advance, and this is not expected to increase the risk of TRALI. A study sponsored by the U.S. Food and Drug Administration, in which COVID-19 convalescent plasma was administered to 20,000 hospitalized patients with COVID-19, showed that COVID-19 convalescent plasma transfusion is safe [19].

To minimize the risks of infections and adverse reactions caused by immunization reactions, the collected plasma is tested, including viral and irregular antibody tests, similar to that of typical blood donations. If a donor has been pregnant or has undergone a blood transfusion, an HLA antibody test will be performed to confirm the negative result and minimize the risk of TRALI caused by the treatment. Before transfusion, cross-matching should be performed. Although the plasma is infused, the medical staff should always be with the subject to carefully monitor changes in their condition. The investigator should provide an explanation to the patients in whom the benefits from convalescent plasma therapy outweigh the risks in his/her judgment and should explain the risks in detail at the time of informed consent. Three months after transfusion, the post-transfusion infection tests specified at the National Centre for Global Health and Medicine, including HBV-DNA quantification, HCV core protein, and HIV antibody assays, will be performed.

## 2. Methods/Design

This trial is an open-label, randomized controlled trial comprising two groups: a convalescent plasma with a standard-care group (convalescent plasma group) and a standard of care group. The plasma administered to patients with COVID-19 randomized in the convalescent plasma group will be that collected and stored in an associated study (study title: A study on the collection of the COVID-19 convalescent plasma and its antibody titer and activity; abbreviation: COVIPLA-D; Institutional Review Board of National Center for Global Health and Medicine; approval No. NCGM-G-003536). Patients with a diagnosis of mild COVID-19 will be included in this trial. The efficacy of convalescent plasma transfusion will be evaluated by comparing the convalescent plasma group to the standard of care group (without convalescent plasma transfusion) with respect to changes in viral load and other measures. This trial was registered with the Japan Registry of Clinical Trials (Clinical Trial Plan Number: jRCTs031200374; https://dbcentre3.jmacct.med.or.jp/jmactr/Default_Eng.aspx; registration date: 24 February 2021).

The latest research protocol is version 2.4, dated 21 October 2021. The principal investigator shall prepare one form of explanation document, consent document, and consent withdrawal form for one research protocol and obtain approval from the NCGM-approved Clinical Research Review Committee. The subject of the clinical research or a surrogate and a witness (a person who attends the consent explanation process for the subject of the clinical research or a surrogate who has the ability to consent but is unable to read the consent document due to visual impairment or other reasons, and who is independent of the conductor of the clinical research) The consent document should be prepared in plain language so that it can be understood by the person who is the subject of the clinical research (a person who is independent of the conductor of the clinical research).

In this multi-center collaborative study, common items other than those specific to each institution (e.g., the name of the principal investigator and contact information for the consultation service) should be described so that the contents of the explanation and consent for the subjects of the clinical research at each institution are consistent. 

Persons engaged in this research (including external parties) shall comply with the “Act on the Protection of Personal Information” (promulgated on 30 May 2003, Law No.57) and related notifications that apply to the protection of personal information, etc. of subjects in clinical research. Those engaged in this research shall not obtain personal information through deception or other wrongful means, shall make the utmost efforts to protect the personal information and privacy of clinical research subjects, and shall not divulge personal information obtained in the course of conducting this research without justifiable reason (the same shall apply even after the person involved has left his/her position).

In addition, those involved in this research must not handle personal information obtained in the course of conducting the research beyond the scope for which consent has been obtained in advance from the subjects of the clinical research.

In handling personal information, the principal investigator must specify the purpose of its use to the greatest extent possible and must keep the personal information accurate and up-to-date to the extent necessary to achieve the purpose of use. In addition, measures necessary to prevent leakage, loss, or damage of personal information and to otherwise appropriately manage personal information shall be taken, and the methods of such measures shall be specifically stipulated as implementation rules.

### 2.1. Trial Inclusion Criteria

- A patient who, or whose legal representative, has provided written consent for the participation of the patient in the trial.
- A hospitalized patient with a confirmed diagnosis of COVID-19 based on a reverse transcription-PCR (RT-PCR) or loop-mediated isothermal amplification (LAMP) assay, antigen test, or other means.
- A patient who met all of the following criteria upon admission to a hospital:
  ○ Patients who can begin receiving study treatment within 5 d after onset.
  ○ Patients with SpO_2_ ≥ 95% on room air.
  ○ Patients aged ≥ 40 years or having at least one of the following underlying diseases: renal impairment, chronic obstructive pulmonary disease, cardiovascular disease, cerebrovascular disorder, malignant tumor, obesity, diabetes mellitus, hypertension, and an immunosuppressive state.
- A patient aged at least 20 years at the time of informed consent.
- A patient who has been infected with SARS-CoV-2 for the first time.
- Our inclusion criteria have been chosen to ensure that the patients are appropriately and ethically included in our study, have a milder form of the disease, are in the appropriate age group, and do not already have SARS-CoV-2 antibodies at the point of enrollment.

### 2.2. Trial Exclusion Criteria

- A patient who is pregnant or breastfeeding.
- A patient who would not undergo a blood transfusion because of their religious beliefs.
- A patient who is participating in an intervention study for the treatment of COVID-19.
- A patient who has been vaccinated against SARS-CoV-2.
- A patient who has already undergone convalescent plasma transfusion.
- A patient with a history of allergy to a blood product.
- A patient with a deficiency in a plasma protein, such as IgA.
- A patient with New York Heart Association class III or IV heart failure.
- A patient whom the principal investigator, investigator, or sub-investigator judged to be ineligible for other reasons.

These exclusion criteria will help ensure the safety of study subjects, that the products used are acceptable to the study subjects, and that the evaluation of the efficacy of our planned intervention is not influenced by other SARS-CoV-2 treatments or vaccination.

### 2.3. Intervention

The intervention in this trial will be injectable COVID-19 convalescent plasma. This product will be the fresh frozen plasma collected by apheresis to remove most of the white blood cells. After being thawed, the liquid appears yellow to yellow-brown, sometimes cloudy because of lipids in the plasma. This product contains an anticoagulant solution (Acid Citrate Dextrose [ACD]-A solution) derived from apheresis. The collected plasma will be aliquoted in a volume of 100 mL; approximately 1.6 to 2.0 g (71 to 78 mEq) of sodium is present in 100 mL [20]. The neutralizing activity of purified IgG from convalescent plasma against SARS-CoV-2 will be evaluated using a previously described in vitro cell-based assay [18]. The neutralizing activity of convalescent plasma will be expressed as total neutralizing units (NU) by purified IgG neutralizing activity, the total amount of human IgG in the plasma, and the total volume of plasma [18]. The activity of the convalescent plasma will be classified into four stages (Supplementary Information S1).

- Grade A: Potent neutralizing activity. The total neutralizing capacity of 200 mL of plasma is ≥18,000 NU (Supplementary Information S2).
- Grade B: Moderate neutralizing activity. The total neutralizing capacity of 200 mL of plasma is ≥9000 but <18,000 NU.
- Grade C: Mild neutralizing activity. The total neutralizing capacity of 200 mL of plasma is ≥4500 but <9000 NU.
- Grade X: Slight neutralizing activity. The total neutralizing capacity of 200 mL of plasma is <4500 NU.

In this trial, there are no required or restricted concomitant drugs or therapies, but other drugs are used as an investigational intervention for COVID-19.

### 2.4. Randomization and Blinding

Subjects will be randomized to the convalescent plasma group or the standard of care group at a ratio of 1:1 by the randomization system of the Electronic Data Capture (EDC) or a randomization system created separately from the EDC. The randomization program will be registered on the randomization system in advance. Detailed procedures for randomization will be specified separately in the procedure manual (outside the scope of review by the certified review board). The purpose of this trial is to evaluate whether convalescent plasma therapy should be added to usual care, and it will be difficult to conduct blind treatment, considering the invasiveness in regards to the subjects and effects on the standard of care. For clinical improvement as secondary endpoints, blinding could be made possible if independent persons assess it. However, many other parameters will be collected regardless of whether they are blinded or not, and unblinding is expected to have a negligible impact. Therefore, treatment will not be blinded in this trial.

Randomization will be stratified by the following factors:

- Age (≥60 or <60 years old).
- The number of days from the day of onset (set as day 0) until the scheduled day of convalescent plasma transfusion (≤3 or ≥4 d).
- Trial site.

### 2.5. Primary and Secondary Endpoints

The primary endpoint of the trial will be the time-weighted average changes in the SARS-CoV-2 virus load in nasopharyngeal swabs from day 0 to days 3 and 5. It is hypothesized that the intervention should result in a decrease in the viral load in the convalescent plasma group up to day 5. This endpoint (change in viral load) has been used as an index of therapeutic effect in several previous studies. 

The secondary endpoints are shown in Table 1.

### 2.6. Dosage and Administration

After consent is obtained from a subject, the convalescent plasma will be administered intravenously during the period between the day of onset (set as day 0) and day 5. It will be infused intravenously at 40 mL/h for the first 15 min after the start of infusion. If no adverse event is observed during the infusion, it will be continued at 100 mL/h. The infused volume will be dependent upon the following.

As one course of therapy, the infused volume of the plasma is 200 mL for Grade A, 400 mL for Grade B, and 800 mL for Grade C. When the volume of 800 mL is infused, it will be divided into 2 and 400 mL will be infused in a 24 h interval.

Plasma with blood types A, B, and O must be administered to the subjects with the same blood type. However, according to the Guidelines for the Use of Blood Products issued by the Ministry of Health, Labour and Welfare [20], plasma with blood type AB can be administered to subjects with blood types A, B, and O, unless there is plasma with the same blood type.

### 2.7. Treatment and Observation Periods (Including Follow-Up)

The Grade A and B convalescent plasma will be administered in a day, and the Grade C convalescent plasma will be administered in 2 d. The subjects will be observed according to the schedule shown in Table 2.

### 2.8. Observation and Test Parameters

- Characteristics of each subject:
  ○ Date of birth (age), sex, nationality/race, smoking history, complications, prior medical history, history of the current disease, and pregnancy status (premenopausal female subjects should undergo a pregnancy test).
  ○ Information on hospitalization (dates of admission and discharge).
  ○ Body height and weight.
  ○ Background data related to COVID-19 and overseas travel history.
- Physical findings:
  ○ Status of supplemental oxygen and the use of mechanical ventilation.
  ○ Physical conditions will be examined by inspection, palpation, auscultation, and percussion.
- Vital signs:
  ○ Level of consciousness
  ○ Body temperature (°C)
  ○ Blood pressure (mmHg)
  ○ Pulse rate (beats/min)
  ○ Respiratory rate (breaths/min)
  ○ SpO_2_ (%)
- Clinical condition:
  The clinical condition of each subject will be assessed according to the following:
  ○ Death
  ○ Hospitalization and the use of invasive mechanical ventilation or ECMO.
  ○ Hospitalization and the use of noninvasive mechanical ventilation or a high-flow oxygen device.
  ○ Hospitalization and supplemental oxygen requirement.
  ○ Not requiring hospitalization or supplemental oxygen but requiring the continuation of treatment (for COVID-19-related or other diseases).
  ○ Not requiring hospitalization, supplemental oxygen, or the continuation of treatment.
  ○ Not requiring hospitalization, but requiring the limitation of activities and/or oxygen therapy at home.
  ○ Not requiring hospitalization or the limitation of activities.
- Laboratory tests (Table 3)
- Imaging
- Plain chest X-ray

The appropriateness of the incorporated participants and the data collected were monitored on a regular basis to ensure quality.

### 2.9. Study Schedule

The study schedule is shown in Table 2. 

### 2.10. Participation and Follow-Up Periods

The subjects in both the convalescent plasma and standard of care groups will be followed up for up to 28 d to detect any adverse events. In addition, the convalescent plasma group will be examined for post-transfusion infections on day 90. The observation period for the trial will run from the day of registration of the first subject until 31 March 2022 (scheduled).

### 2.11. Potential Benefits and Risks of the Study Drug

See Supplementary Information S3.

### 2.12. Sample Size Calculation

The trial was designed to enroll 200 patients. We calculated that this sample size would provide 90% power to detect a between-group log viral load difference of 0.5 (SD 1.1) at the 0.05 (two-sided) level of significance, accounting for several drop-out subjects.

### 2.13. Statistical Analysis Plan

See Supplementary Information S4.

### 2.14. Safety Evaluation

See Supplementary Information S5.

### 2.15. Data Monitoring Committee

The committee was established to neutrally evaluate interim data during the study period and to provide appropriate advice and recommendations to ensure the safety of subjects and the ethical and scientific validity of the study. In fact, it was not conducted due to a lack of progress in incorporation.

In addition, the committee periodically evaluated the following:

1. Changes over time in the treatment regimen in both treatment groups
2. Relationship between the quality of the study drug (Grades A to C) and safety

### 2.16. Data Management Team

The Director of the Joint Center for Researchers, Associates and Clinicians (JCRAC) Data Center designated a person to be in charge of data management tasks, and the data management staff carried out the tasks specified in the data management plan.

## 3. Discussion

COVID-19 is a significant public health concern because of its high associated morbidity and mortality. As of December 2020, the therapeutic agents approved for COVID-19 in Japan were limited to two drugs: remdesivir, an antiviral drug, granted a Special Approval for Emergency on 7 May 2020, and dexamethasone, which has an anti-inflammatory effect. Several case reports and clinical studies of convalescent plasma therapy have been reported [21,22,23,24,25,26,27,28,29], but the results have been conflicting. This could have occurred because the neutralizing antibody titer in the COVID-19 convalescent plasma administered was not measured in most studies, and there appears to be an association between a higher antibody index and severe COVID-19 [30]. Thus, in this trial, subjects will receive plasma with high neutralizing activity after it is determined whether the convalescent plasma from each donor can inhibit infection when susceptible living cells are exposed to infectious novel coronaviruses. The objective of this trial is to evaluate the efficacy of the convalescent plasma collected from donors who recovered from COVID-19.

There have been several important findings in nonclinical and clinical studies of relevance to this study. Purified IgG from convalescent plasma inhibited the cytotoxicity and replication competence of SARS-CoV-2 in an in vitro cell infection system. At a high concentration, it can completely inhibit SARS-CoV-2 infection at the single-cell level [18]. In a hamster model representing an in vivo cell infection system, the administration of convalescent plasma inhibited viral replication in the lung [31]. However, several RCTs of convalescent plasma therapy have already been conducted in countries outside Japan, but no conclusion has been reached with respect to the efficacy of convalescent plasma therapy, which is likely in part because of the heterogeneity of the types of target patients, interventions, and endpoints among trials [32]. Furthermore, it is well known that SARS-CoV-2 viruses have undergone multiple mutations since their appearance in 2019, resulting in changes in virulence that affect disease severity worldwide. Although it was reported that convalescent plasma therapy has led to a significant decrease in mortality rates among SARS-CoV-2 patients, the efficacy of the therapy would be affected by the degree of severity caused by multiple mutations of SARS-CoV-2 variants [25]. Further studies, such as on the early administration of convalescent plasma containing appropriate antibodies, will be needed to demonstrate the efficacy of convalescent plasma therapy. 

### Limitations of the Trial

As described above, the proposed trial has the potential to prevent patients with mild COVID-19 from developing a more severe illness, but there will be some limitations to this trial. First, for subjects receiving standard treatments for COVID-19 that will not be withheld as part of the trial, it may be difficult to disentangle the effectiveness of convalescent plasma therapy on the outcomes of subjects from one of the standard treatments. Second, although the number of persons vaccinated against SARS-CoV-2 has increased in Japan, the potential for beneficial effects of convalescent plasma therapy for vaccinated subjects remains unknown. In this trial, subjects vaccinated against SARS-CoV-2 will be excluded; therefore, the number of subjects who could take part in the trial will decrease over time. Third, casirivimab/imdevimab and sotrovimab, which are anti-SARS-CoV-2 monoclonal antibody treatments, and molnupiravir, an antiviral drug, were approved in Japan from July to December 2021 to prevent the development of symptomatic COVID-19, approximately 5–10 months after the first patient enrolled in this trial in February 2021. Because the target population groups of casirivimab/imdevimab, sotrovimab, and molnupiravir therapy overlap with that of this trial, this could cause difficulties when enrolling subjects. Taking these limitations into consideration and closely observing the developing trends of novel drugs and therapies, issues regarding subject enrollment should be reconsidered in the future. Nonetheless, improvements shown by our trial may lead to the expansion of the use of convalescent plasma therapy.

## Figures and Tables

**Table 1:** Secondary endpoints of the trial.

Objective	Endpoint	Rationale for the Endpoint
Prevention of mechanical ventilation or death.	Use of mechanical ventilation or death by days 14 and 28.	Prognosis is an endpoint that is not subjective.
Prevention of death.	Mortality on days 14 and 28.	Prognosis is an endpoint that is not subjective.
Prevention of the need for supplemental oxygen use.	Percentage of subjects who used oxygen on days 3, 5, 7, 14, and 28.	It is an endpoint that is not subjective.
To assess the shortening of the duration of symptoms (the time to clinical improvement).	Clinical improvement is defined as the first day a subject meets one of the three categories on the ordinal scale shown below: Not requiring hospitalization or supplemental oxygen and not requiring continuation of treatment. No hospitalization is needed but requires the limitation of activities and/or oxygen therapy at home. No hospitalization and no limitation of activities.	Clinical improvement is related to efficacy.
To assess clinical improvement on days 3, 5, 7, 14, and 28 in subjects given the convalescent plasma.	Clinical improvement on days 3, 5, 7, 14, and 28 (on an 8-point scale)	Clinical improvement is related to efficacy.
Time to improvement on the National Early Warning Score, UK (NEWS)	Time to discharge from the hospital or the maintenance of NEWS ≤2 for 24 h (whichever occurs first) NEWS on days 3, 5, 7, 14, and 28.	Clinical improvement is related to efficacy.
Decrease in the viral load in the convalescent plasma group after convalescent plasma transfusion.	Time-weighted average change and the numerical change in the SARS-CoV-2 virus load in nasopharyngeal swabs from day 0 to each day of assessment.	Change in the viral load has been used as the index of the therapeutic effect in many studies.
To assess safety after convalescent plasma transfusion.	Occurrence of adverse events.	It is necessary to evaluate safety.
To screen and identify variants.	Determine if variants are present in nasopharyngeal swab samples on day 0	Variants are related to efficacy because it has been reported that variants may reduce the antiviral activity of neutralizing antibodies.

**Table 2:** Study schedule.

Activities	Admission		Day 0 (Day of Transfusion) ^i^		Day 1 (1 d after Transfusion)	Day 3 (3 d after Transfusion)	Day 5 (5 d after Transfusion)	Day 7 (7 d after Transfusion)	Day 14 (14 d after Transfusion)	Day 21 (21 d after Transfusion)	Day 28 (28 d after Transfusion)	Day 90 (90 d after Transfusion)	Discontinuation of the Study
			Before Trans- Fusion	3 h after Start of Trans-Fusion ^j^									
Acceptable time window ^a^	−3 to 0		Reference day		+1	±1	±1	±1	±3	±3	±3	+30	–
Informed consent	X												
Confirmation of eligibility		X ^k^											
Registration and randomization of a subject		X											
Characteristics of a subject		X ^k^											
Plasma transfusion ^h^			X		X ^b^								
Vital signs ^d^ and clinical condition ^c^			X	X ^h^	Once daily during hospitalization and days 3, 5, 7, 14, 21, and 28 after discharge								X
Physical findings ^d^		X ^k^	X	X ^h^	X	X	X	X	X	X	X		X
Pregnancy test ^l^		X ^k^											
Collection of swabs (2 sticks) ^m^			X		X	X	X	X	X	X	X		X ^e^
Blood test (biochemistry, complete blood count, and coagulation) ^m^		X ^k^			X	X	X	X	X	X	X		
Blood test (blood type)		X ^k^											
Blood test (cross-match) ^h^		X											
Blood test (infection screening) ^f^		X ^k^											
Blood test (post-transfusion infection test) ^g,h^												X	
Storage of plasma ^m^			X		X	X	X	X	X	X	X		
Storage of serum ^m^			X		X	X	X	X	X	X	X		
Radiography (chest X-ray)		X ^k^											
Concomitant drugs ^c^			X		Every day during hospitalization and days 3, 5, 7, 14, 21, and 28 after discharge								X
Adverse events ^c^				X ^h^	Every day during hospitalization and days 3, 5, 7, 14, 21, and 28 after discharge							X ^h^	X

^a^ If the days overlap because of the acceptable time window, it is not allowed to collect the data for 2 d on the same day (e.g., it is not allowed to collect the data for both day 1 and day 3 on day 2). ^b^ When Grade C plasma is used, it will be infused on day 1. ^c^ When a subject has been discharged, the data can be collected by phone on or after day 7. ^d^ When a subject has been discharged, it is allowed to not collect data on or after day 7. ^e^ This should be performed if a subject discontinues the study on and before day 14. ^f^ Similar to tests for infectious diseases conducted at registration, HBsAg, HBsAb, HCVAb, HIV-1/2Ab, Syphilis-RPR/TPHA, and HTLV-1 Ab should be measured. ^g^ Similar to the post-transfusion infection test, HBV-DNA quantification, HCV core protein, and HIV-1/2Ab assays should be performed (the test should also be performed in a subject who has stopped transfusion prematurely). ^h^ These data will be collected in the convalescent plasma group. ^i^ The date of transfusion scheduled at the time of randomization is day 0 in both the convalescent plasma group and the standard of care group. ^j^ When Grade C plasma is used, the data to be collected 3 h after the start of transfusion on day 0 should be collected both on days of first and second transfusions. ^k^ The results of the tests as the regular practice before informed consent can be used. ^l^ It is performed in premenopausal female subjects. ^m^ When a subject has been discharged, it is allowed to not collect samples on or after day 14.

**Table 3:** Laboratory tests performed as part of the trial.

Hematology	Hemoglobin, Hematocrit, White Blood Cell Count with Differential, and Platelet Count
Coagulation	APTT, PT-INR, and D-Dimer
Blood biochemistry	Albumin, AST, ALT, bilirubin, CRP, blood glucose, urea nitrogen, creatinine, LDH, creatine kinase, potassium, and sodium
Infection screening	HBsAg, HBsAb, HCVAb, HIV-1/2Ab, Syphilis-RPR/TPHA, and HTLV-1Ab.
Pregnancy test	Urine or blood (HCG)
Blood type and cross-match	Blood type: A/O/B/AB, Rh +/−; cross-match: compatible/incompatible
SARS-CoV-2 viral load	Nasopharyngeal swabs
Samples for storage	Serum (1.5 mL) and plasma (1.5 mL)
Post-transfusion infection test	HBV-DNA quantification, HCV core protein, and HIV-1/2Ab

## Data Availability

Data are available from the principal investigator on reasonable request.

## Supplementary Materials

The following supporting information can be downloaded at: https://www.mdpi.com/article/10.3390/life12060856/s1. Refs. [19,33,34,35] are cited in the Supplementary Materials.

## Abbreviations

COVID-19	coronavirus disease 2019
ECMO	extracorporeal membrane oxygenation
EDC	Electronic Data Capture
MERS	Middle East respiratory syndrome.
NIH	National Institute of Health
RCTs	randomized controlled trials
SARS	severe acute respiratory syndrome
WHO	World Health Organization
