# Supplementary material for: A Multi-Center, Open-Label, Randomized Controlled Trial to Evaluate the Efficacy of Convalescent Plasma Therapy for Coronavirus Disease 2019: A Trial Protocol (COVIPLA-RCT)
Source: Life (Basel). 2022 Jun 8;12(6):856. doi: 10.3390/life12060856 (PMC9225318; doi:10.3390/life12060856)
Supplement: Supplementary file 1 [file life-12-00856-s001.zip › life-1742310-supplementary.pdf]

Supplementary Information

Supplementary Information S1

As of December 22, 2020, antiviral activity and IgG levels have been measured in the plasma of 36 donors. The number of donors whose plasma has been classified according to each grade was as follows: grade A, three (8.3%); grade B, five (13.9%); grade C, 12 (33.3%); and grade X, 16 (44.4%). Grade A plasma from one donor corresponded to the convalescent plasma of two subjects. Grades B and C plasma from one donor corresponded to the convalescent plasma for one subject and one-half subject, respectively. Therefore, convalescent plasma for 17 subjects was obtained from 36 donors.

Supplementary Information S2

Purified IgG derived from the standard donor at 100 μg/mL completely inhibited SARS-CoV-2 infection at the cellular level in an in vitro cell infection system. The antiviral activity per unit of IgG in the plasma at that was defined as a neutralizing capacity of 10 U/mg. Based on the body fluid volume of humans, it is considered necessary to administer a total of 12,000 neutralizing units (NU) to achieve a similar complete antiviral activity in the body. The dose calculated by multiplying 12,000 NU by a safety factor of 1.5 has been set as the reference dose.

Supplementary Information S3

Potential benefits of the study drug

By receiving convalescent plasma therapy, patients who participated in this trial could experience a reduced risk of worsening or severe pneumonia and respiratory symptoms caused by SARS-CoV-2. In addition to direct benefits to subjects, if this trial exhibits the efficacy of the convalescent plasma therapy, it will provide important evidence for the

treatment of COVID-19. The findings of this trial may be used to provide benefits to society in the future.

Potential risks of the study drug

The convalescent plasma in this trial may have the same risks as the adverse reactions to fresh frozen plasma because it undergoes the same testing, processing, and storage as the plasma collected by apheresis donations. If a donor is affected by an unknown infection that is currently difficult to screen, a subject who has received plasma may acquire the infection. Although the donors will be interviewed according to the screening procedures used by the Japanese Red Cross Society, the risk of infection in this trial could be higher than that of standard blood transfusion because some screening criteria have been changed in this trial, allowing the acceptance of convalescent plasma from patients with COVID-19 or diabetes mellitus. In addition, adverse reactions, such as hypersensitivity, shock, anaphylaxis, and multi-organ failure, may occur because of immunization and antigen–antibody reactions. If a donor has been pregnant before or has undergone a blood transfusion, HLA antibodies could be present in the blood product, which could increase the risk of TRALI after administration of the plasma product. However, persons who tested positive for HLA antibodies are excluded from the donors in advance, and this is not expected to increase the risk of TRALI. A study sponsored by the U.S. Food and Drug Administration, in which COVID-19 convalescent plasma was administered to 20,000 hospitalized patients with COVID-19, showed that COVID-19 convalescent plasma transfusion is safe.[1]

To minimize the risks of infections and adverse reactions caused by immunization reactions, the collected plasma is tested, including viral and irregular antibody tests, similar to that of typical blood donations. If a donor has been pregnant or has undergone a blood transfusion, an HLA antibody test will be performed to confirm the negative result and

minimize the risk of TRALI caused by the treatment. Before transfusion, cross-matching should be performed. Although the plasma is infused, the medical staff should always be with the subject to carefully monitor changes in their condition. The investigator should provide an explanation to the patients in whom the benefits from convalescent plasma therapy outweigh the risks in his/her judgment and should explain the risks in detail at the time of informed consent.

Three months after transfusion, the post-transfusion infection tests specified at the National Center for Global Health and Medicine, including HBV-DNA quantification, HCV core protein, and HIV (1, 2) antibody assays, will be performed.

Reference

1. Joyner MJ, Bruno KA, Klassen SA, et al. Safety update: COVID-19 convalescent plasma in 20,000 hospitalized patients. *Mayo Clin Proc*. 2020;95(9):1888-1897.

Supplementary Information S4

Statistical analysis plan

Analysis sets

Efficacy analysis will be performed in the modified intention-to-treat (MITT) analysis set, and safety analysis will be performed in the safety analysis set.

- MITT analysis set: This includes all randomized subjects who do not meet any of the following criteria:
    1. A subject with no measured value for the efficacy endpoint.
    2. A subject who does not meet the criteria for registration.
    3. A subject who has not undergone convalescent plasma transfusion as specified (for 1 or 2 d).

- Safety analysis set: This includes all randomized subjects but excludes those for whom no safety assessment was performed.

Planned number of registered subjects

The planned sample size for the entire trial is 200 participants. The rationale for the sample size included the power of the trial and clinical considerations.

The convalescent plasma used in this trial is plasma that has been collected and stored in COVIPLA-D. Because the data obtained after transfusion of the above plasma in patients with COVID-19 at this center are limited to patients with moderate disease, we first focus on the change in Ct value for patients with moderate disease. In the eight patients with moderate disease who underwent convalescent plasma transfusion, the Ct values for viral loads were 30.5 (standard deviation [SD] = 6.8) at transfusion, 34.8 (SD = 5.5) 3 d after transfusion, and 40.0 (SD = 3.5) 7 d after transfusion. The changes at each time point were 4.2 (SD: 5.1) and 7.7 (SD = 7.1), respectively. However, changes in the Ct value up to 5 d after admission for patients with moderate COVID-19 were 2.7 (SD = 2.2) in 3 patients at this center and 2.3 (SD = 1.5) were reported by Xu et al.[1] (values were approximated from the figure in the article). The change until day 7 was 2.9 (SD = 9.5) at this center, approximately 4.5 (SD = 4.5) according to Liu et al.,[2] and approximately 3.5 (SD = 2.5) in the report by Xu et al.[1] (the values were approximated from the figures in the articles).

Thus, the differences in the changes in the Ct values between patients with moderate disease who underwent convalescent plasma transfusion at this center and the untreated patients in prior studies were 1.6 and 1.9 on day 5 and 3.2, 3.8, and 4.8 on day 7 of hospitalization. The SD for the change between the patient groups at this center was 4.7. Although the effect of convalescent plasma transfusion was observed on day 7 of

hospitalization in patients with moderate disease, it is expected to achieve the effect of plasma antibodies by day 5 of hospitalization in patients with mild disease in this trial, based on the results of previous studies.

Although it is difficult to accurately estimate the difference between groups until day 5 in patients with mild disease, it is estimated to be a halfway value within the range of the differences between groups. Assuming that the difference between groups is 2.0, with an SD of 4.6, the necessary sample size to detect the efficacy at a power of 90% with an alpha error of 5% is 192 if subjects are randomized to the convalescent plasma group or the standard of care group at a ratio of 1:1. To allow for some dropouts, the planned number of participants has been set at 200. Weinreich et al.[3] evaluated the time-weighted average change in the viral load ($\log_{10}$ copies/mL) in the plasma antibody and placebo groups until day 7, and the difference between the groups was 0.41 (SE = 0.15). Weinreich et al. included subjects who were admitted to the hospital within 7 d of onset. We expect to obtain a slightly larger effect size in this trial than that of Weinreich et al. because the difference between groups will be evaluated until day 5 in this trial (subjects within 5 d after onset will be included in this trial). Assuming that the difference in the viral load ($\log_{10}$ copies/mL) between groups is 0.5 (SD = 1.1), it is ensured that the power is approximately 90%. The SD will be calculated in the combined group in the interim analysis, and if the variation is larger than expected, the sample size may be increased if feasible.

To attain the planned sample size, the planned number of subjects will be set for each trial site. However, the planned number of subjects in each trial site is an expected number, and it is allowed to exceed this number in a trial site. The principal investigator should manage the number of subjects registered at each trial site such that the total number of subjects does not exceed the planned sample size in the trial. If it is necessary to increase the planned sample size in the entire trial, the principal investigator should obtain approval from

the certified review board in advance and submit the notification to the Minister of Health, Labour and Welfare.

Level of significance

The two-tailed significance level for this trial will be 5%.

Statistical analyses for efficacy

- Analysis of the primary endpoint

In the MITT analysis set, the change in SARS-CoV-2 virus load from day 0 to days 3 and 5 will be evaluated. The decrease in viral load at the above time points will be calculated using the trapezoidal rule and will be divided by the number of days until up to day 5 to calculate the time-weighted average change. A comparison will be made between the treatment groups by analysis of covariance with respect to the time-weighted average change. The factors in the model will be the treatment group, viral load at baseline, age, and the number of days from onset until admission to the hospital.

As a secondary analysis, subgroup analysis will be performed for age (≥60 or <60 years) and the number of days after onset (≤3 or ≥4 d). Moreover, subgroup analysis for variants will be performed in groups with and without variants, and the groups with and without a variant will be derived from Brazil, South Africa, the Philippines, and Japan (E484K).

- Analyses of the secondary endpoints
    - Prevention of mechanical ventilation or death and use of oxygen. For these three endpoints, the time to each event will be analyzed. The Kaplan–Meier curve will be calculated, and a comparison will be made between the two treatment groups using

the log-rank test. The hazard ratio in the convalescent plasma group relative to the standard of care group and its 95% confidence interval (CI) will be calculated. For the prevention of mechanical ventilation or death, and death, the probability of the above events will be calculated on days 14 and 28. For the use of oxygen, the percentage of subjects who are using oxygen will be calculated on days 3, 5, 7, 14, and 28.

- Time to clinical improvement. The Kaplan–Meier curve will be calculated for the time to any of the following conditions: "not requiring hospitalization or supplemental oxygen and not requiring the continuation of treatment," "no hospitalization but requiring the limitation of activities and/or oxygen therapy at home," and "no hospitalization and no limitation of activities." A comparison will be made between the two treatment groups using the log-rank test. The hazard ratio in the convalescent plasma group relative to the standard care group and its 95% confidence interval will be calculated.
- Clinical improvement in subjects administered convalescent plasma. For the clinical improvement assessed on an eight-point scale, the number and percentage of subjects with each score on days 3, 5, 7, 14, and 28 will be calculated, and a comparison will be conducted between the treatment groups using the Mantel test. For the scores on each day, the odds ratio and 95% CI will be calculated using the proportional odds model. The distribution of changes in the score from day 0 to days 3, 5, 7, 14, and 28 will be calculated.
- Time to improvement using the National Early Warning Score, UK (NEWS). For the time to discharge from the hospital or the maintenance of NEWS ≤ 2 for 24 h (whichever occurred first), the Kaplan–Meier curve will be calculated, and a comparison will be made between the two treatment groups using the log-rank test.

The hazard ratio in the convalescent plasma group relative to the standard care group and its 95% CI will be calculated.

  - Viral load in the convalescent plasma group on each assessment day. For the viral load and change in viral load from day 0 to each day of assessment, the mean and SD) will be calculated. The same analysis as that for the primary endpoint will be performed for the time-weighted average changes in the viral load on days 7 and 14. The least-square means of changes on each day of assessment and the difference between groups will be calculated using the mixed-effect model for repeated measures.

Statistical analyses for safety

The following analyses will be performed in the safety analysis set.

- Adverse events. For each adverse event, the seriousness, severity, intervention, outcome, and causal relationship to convalescent plasma will be summarized, and the number of subjects with the adverse event will be calculated.
- Vital signs and laboratory parameters. For vital signs and laboratory parameters, the summary statistics (mean, SD, minimum, maximum, and median) and change from day 0 will be calculated for each time point.

Further information regarding the safety analysis for this study is provided in Supplementary Information S3.

Discontinuation criteria for the trial

- Criteria for withdrawing individual subjects from the trial.

If a participant met one of the following criteria during the trial, the subject would be withdrawn from the trial. For the subject, the test, observation, and assessment at the discontinuation of the study will be performed, whereas the necessary treatment is continued. The date of discontinuation (the date where the investigator or sub-investigator has decided to withdraw the subject), reasons for discontinuation, intervention, and the clinical course will be recorded on the Electronic Data Capture. If a subject is withdrawn from the trial because of an adverse event, the subject should be followed-up until the event has been resolved. If the subject does not visit the trial site, the investigator or sub-investigator should continue the follow-up by mail, phone, or other means.

- A subject has withdrawn the consent to participate in the trial.
- Participation in the trial has been stopped for subject reasons (such as the change of residence, change of physicians/transfer to another hospital, business, and loss of follow-up).
- It has been found that, after the start of a trial, the subject is ineligible.
- One subject wishes to participate in another intervention study against COVID-19 because of the worsening of the primary disease.
- It has been found that a subject is pregnant.
- A subject is considered not evaluable because of a significant protocol deviation.
- The investigator or sub-investigator considers it difficult to continue the participation of a subject for other reasons.

When convalescent plasma transfusion is discontinued prematurely because of an adverse reaction or other reason and is not completed after the start of transfusion, the patient should be provided with the necessary care and monitored for 24 h after the transfusion is discontinued. The event should be reported as an adverse event or serious adverse event. The remaining plasma will be collected and used in another study (the storage of the sample, in

this case, is specified in the protocol of the study on the collection of the plasma [COVIPLA-D: NCGM-003536]). Subjects who discontinued transfusion prematurely or have been withdrawn from the trial should undergo a post-transfusion infection test on day 90 in the convalescent plasma group, as specified in the protocol.

- Termination or suspension of the trials. If the following occurs, the trial should be temporarily suspended, and the Data Monitoring Committee should discuss whether to continue the trial. If the trial is suspended, the principal investigator should immediately inform the investigator at each trial site regarding the decision, who should cease the registration of new subjects. Trial activities in subjects who have already been registered will continue until the data monitoring committee has made a decision.
    - The risk of participation in the trial is thought to outweigh the benefits because of safety issues.
    - Serious non-compliance with laws, regulations, and protocols has been observed.
    - The certified review board expressed a negative opinion.
    - The Minister of Health, Labour and Welfare requested or recommended the discontinuation.
    - The Data Monitoring Committee decided on discontinuation.

When the Data Monitoring Committee has judged it appropriate to continue the trial as a result of the discussion, the registration of subjects will be resumed. If the Committee judged it appropriate to terminate the trial, the principal investigator should immediately inform the investigators of all trial sites regarding the discontinuation and report it to the head of each trial site and the certified review board. The investigators should inform each subject regarding this to schedule the next visit for post-treatment follow-up.

References

1. Xu T, Chen C, Zhu Z, et al. Clinical features and dynamics of viral load in imported and non-imported patients with COVID-19. *Int J Infect Dis*. 2020;94:68-71.
2. Liu Y, Yan LM, Wan L, et al. Viral dynamics in mild and severe cases of COVID-19. *Lancet Infect Dis*. 2020;20(6):656-657.
3. Weinreich DM, Sivapalasingam S, Norton T, et al. REGN-COV2, a neutralizing antibody cocktail, in outpatients with Covid-19. *N Engl J Med.* 2021;384(3):238-251

Supplementary Information S5

Safety evaluation

Safety endpoints

- Frequencies of adverse events and percentages of subjects with adverse events.

Assessment, records, and analysis of safety endpoints and their timing

The dates of occurrence and resolution, severity, intervention, outcome, seriousness, causal relationship with drugs, and expectedness of an adverse event will be recorded in the medical record and case report form of the subject.

- Criteria for severity (assessed from the start to the end of transfusion in the convalescent plasma group).

The severity will be assessed according to the following criteria.

[1] Mild: Transfusion can be continued without treatment.

[2] Moderate: Transfusion can be continued with some treatment.

[3] Severe: Transfusion has been or should be stopped.

- Criteria for seriousness

Seriousness will be assessed according to the following criteria.

[1] Serious

① Death

② Conditions that could result in death.

③ Conditions that require hospitalization or prolongation of hospitalization for treatment.

④ Disability

⑤ Conditions that could result in disability.

⑥ Any serious conditions other than ① through ⑤ above.

⑦ Congenital diseases or abnormalities in the next generation.

[2] Non-serious

Conditions other than the "serious" conditions above.

Procedures for the collection, records, and reports of data on disease or similar information

The investigator or sub-investigator will record all of the adverse events that have occurred in subjects during participation in the trial and judge the causal relationship to the trial. Adverse events were recorded from day 0 (the start of transfusion on day 0 in the convalescent plasma group). Existing clinical findings before day 0 (just before the start of transfusion in the convalescent plasma group) will be regarded as the underlying diseases or comorbidities and will not be recorded as adverse events. Events that occurred after day 28 will not be recorded as adverse events. However, worsening of underlying diseases will be recorded as an adverse event. In the convalescent plasma group, if any infection is confirmed

by the post-transfusion infection test on day 90, it will be recorded as an adverse event. The details are described in the Procedures for Actions Against Adverse Events and Diseases.

Report of disease or similar conditions to the certified review board (a trial with an unapproved drug or off-label use)

If the investigator becomes aware of any condition from [1] to [7] below, he/she should immediately report it to the head of the trial site and the principal investigator within the reporting time frames shown below.

[1] Death

[2] Conditions that could result in death.

[3] Conditions that require hospitalization or the prolongation of hospitalization for treatment.

[4] Disability

[5] Conditions that could result in disability.

[6] Any serious conditions other than [1] through [3] above.

[7] Congenital diseases or abnormalities in the next generation.

The principal investigator will prepare the report in cooperation with the investigator and sub-investigators and share the data with all investigators. Necessary actions, including warnings, should also be taken. Each investigator should report the shared information on the occurrence of disease or similar conditions to the head of the trial site and take action, if necessary.

For unexpected events of death and conditions that could result in death ([1] and [2] above), the principal investigator should report them within 7 d to the heads of the trial sites and the Clinical Research Safety Management Section of the National Center for Global

Health and Medicine (NCGM), and the NCGM Certified Review Board and Minister of Health, Labour and Welfare. The principal investigator should report them within 15 d to the heads of the trial sites and the NCGM Certified Review Board. For unexpected events of the conditions of [3] through [7] above, the principal investigator should report them within 15 d to the heads of the trial sites and the NCGM Clinical Research Safety Management Section, and as the NCGM Certified Review Board and the Minister of Health, Labour and Welfare. The principal investigator will prepare a report to the Minister of Health, Labour and Welfare using the report form of disease or similar conditions on the Japan Registry of Clinical Trials (jRCT).

The occurrence of diseases or similar conditions suspected to be caused by the trial (excluding the events reported according to the methods above) will be reported periodically to the NCGM Institutional Review Board for Clinical Research.

Observation period for subjects after the occurrence of disease or similar conditions Non-serious adverse events considered causally related to the trial will be followed-up whenever possible until the event is resolved or resolving. All serious adverse events should be followed up until the event is resolved or resolving. If the physician registered in this trial judges the event as permanent (e.g., death, disability, and sequela) or lost to follow-up, the follow-up will end.

[Report of disease or similar conditions to the certified review board] Unexpected diseases or similar conditions in the double frames as reported to the Minister of Health, Labour and Welfare.

**Trial category**	**Expectedness**	**Seriousness of diseases or similar conditions**	**Reporting time frame**

Trial with an unapproved drug or off-label use	Unexpected	Death<br>Conditions that could result in death	7 d
	Expected	Death<br>Conditions that could result in death	15 d
	Unexpected	Conditions that require hospitalization or the prolongation of hospitalization for treatment<br>Disability<br>Conditions that could result in disability<br>Any serious conditions other than the above, death, or conditions that could result in death<br>Congenital diseases or abnormalities in the next generation	15 d
Occurrence of diseases or similar conditions suspected to be caused by the trial (excluding the events reported according to the above)			Periodic reports
